# Resonance-Enhanced Multiphoton Ionization Spectrum and Computational Study of 2‑Cyclopenten-1-one in Its *T*_1_(*n*, π*) State

**DOI:** 10.1021/acs.jpca.5c02355

**Published:** 2025-06-16

**Authors:** Alexander W. Narkiewicz-Jodko, Sean W. Parsons, Hansuja Chaurasia, Stephen L. Coy, Stephen Drucker

**Affiliations:** † Department of Chemistry and Biochemistry, 14747 University of Wisconsin-Eau Claire, 105 Garfield Avenue, Eau Claire, Wisconsin 54701, United States; ‡ 2167 Massachusetts Institute of Technology, 77 Massachusetts Avenue, Cambridge, Massachusetts 02139, United States

## Abstract

The 2-cyclopenten-1-one molecule (2CP) is a cyclic conjugated enone that participates in a variety of photochemical reactions. Prior computational work indicates that the *T*_1_(*n*, π*) excited state of 2CP mediates relaxation processes that can lead to photoproducts. In this paper, we report the *T*_1_(*n*, π*) ← *S*_0_ vibronically resolved spectrum of 2CP, recorded in a supersonic free-jet expansion using resonance enhanced multiphoton ionization (REMPI) detection. The REMPI spectrum covers the region extending to +900 cm^–1^ with respect to the *T*_1_(*n*, π*) ← *S*_0_ origin band at 25,956 cm^–1^. Vibronic analysis of the REMPI spectrum yielded fundamental frequencies for eight vibrational modes in the *T*_1_(*n*, π*) state, including four modes that could not be observed in the jet-cooled phosphorescence excitation spectrum we reported previously. We observe that the out-of-plane, but not in-plane modes, undergo dramatic frequency reduction upon electronic excitation. This distinction sharpened our understanding of the π* ← *n* chromophore. We used the measured *T*_1_(*n*, π*) fundamental frequencies to test a computational method, termed coupled-cluster/density functional theory (CC/DFT) hybrid, that was developed by Puzzarini and Barone for predicting spectroscopic properties of medium-sized molecules (up to about 10 heavy atoms). In our implementation of CC/DFT, we employed the unrestricted coupled cluster singles and doubles with perturbative triples (CCSD­(T)) ab initio technique to calculate harmonic frequencies of the *T*_1_(*n*, π*) state of 2CP. We used second-order vibrational perturbation theory (VPT2) to obtain anharmonic corrections, in conjunction with anharmonic force constants computed using unrestricted DFT. The calculation predicts *T*_1_(*n*, π*) fundamental frequencies that deviate by only 8 cm^–1^, on average, from those measured in the REMPI spectrum. We used the CC/DFT results as a reference to evaluate the performance of more economical hybrid methods for predicting excited-state fundamentals. These methods incorporate equation-of-motion excitation energies coupled cluster singles and doubles (EOM-EE-CCSD) or time-dependent density functional theory (TDDFT) to calculate harmonic frequencies. Notably, the economical TDDFT approaches outperform the EOM-EE-CCSD ab initio technique in this application.

## Graphical abstract

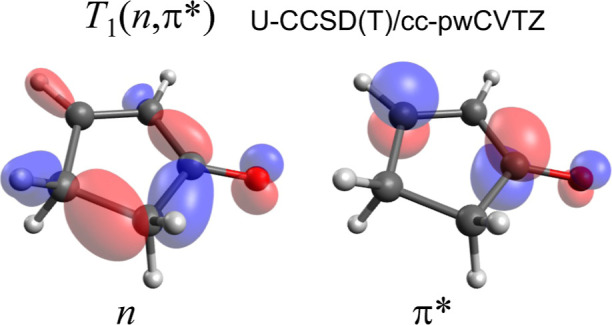

## Introduction

The 2-cyclopenten-1-one molecule (2CP, Figure 1) is a simple conjugated enone with interesting photophysical and photochemical properties. Ultraviolet excitation of 2CP in solution prepares the *S*_1_(*n*, π*) state, which relaxes rapidly by intersystem crossing (ISC) to the lowest triplet excited states, *T*_1_(*n*, π*) and *T*_2_(π, π*).^1–3^ These triplet states mediate much of the photochemistry, including dimerization, cycloaddition, and reductions.^1^ An alternate fate of the triplet states is ISC to high vibrational levels of the electronic ground state, *S*_0_. However, in rigidly planar cyclic enones such as 2CP, a relatively slow nonradiative decay to the ground state leads to long triplet lifetimes and a significant photochemical yield from reactions occurring on a triplet surface.^2,4^

**Figure 1:**
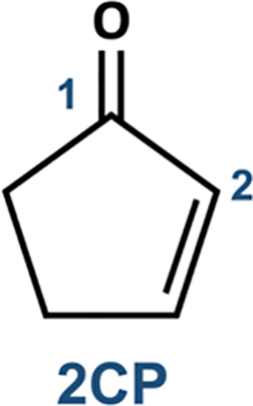
2-Cyclopenten-1-one (2CP). The molecule has a *C*_s_ structure in its ground electronic state, with all heavy atoms coplanar.

When the *T*_1_(*n*, π*) state of 2CP is prepared in the gas phase under isolated-molecule conditions, *T*_1_(*n*, π*) → *S*_0_ phosphorescence can compete favorably with *T*_1_(*n*, π*) ⇝ *S*_0_ ISC, provided the triplet excitation populates a low-lying vibrational state. In 2007, we reported a vibronically resolved phosphorescence excitation (PE) spectrum of the *T*_1_(*n*, π*) ← *S*_0_ band system of 2CP in a supersonic free-jet expansion.^5^ The spectrum was measurable in a region of low vibronic excitation (<500 cm^–1^ above the origin band); the phosphorescence becomes undetectable at higher *T*_1_(*n*, π*) vibrational levels, presumably because of much faster ISC to the ground state.

In this paper, we report a new measurement of the jet-cooled *T*_1_(*n*, π*) ← *S*_0_ band system of 2CP, now extending to +900 cm^–1^ with respect to the origin (0_0_^0^) band. We used resonance enhanced multiphoton ionization (REMPI) spectroscopy to bypass the problem of low quantum yield apparent in the high-energy region of the PE spectrum. In the REMPI experiment, the *T*_1_(*n*, π*) state is detected by ionization promptly following *T*_1_(*n*, π*) ← *S*_0_ photoexcitation, without any time elapsing for nonradiative decay.

Our motivation for studying the *T*_1_(*n*, π*) state of 2CP spectroscopically comes from a series of computational investigations^4,6–8^ of the molecule in its valence excited states. The ultimate computational objective was to describe in detail the photophysical decay pathways following *S*_1_(*n*, π*) ← *S*_0_ optical excitation. In 2001, García-Expósito et al. used the complete active-space self-consistent field (CASSCF) method to determine equilibrium structures on the *S*_1_(*n*, π*), *T*_2_(π, π*), and *T*_1_(*n*, π*) surfaces.^4^ They found that all of the ring atoms are coplanar in the (*n*, π*) excited-state equilibrium structures, as in the ground state. The ^3^(π, π*) state has its minimum at a ring-twisted geometry, according to these calculations. In 2012, Fišanová et al. optimized the geometries^7^ using density functional theory (DFT) and second-order Møller–Plesset perturbation theory (MP2), obtaining results similar to the CASSCF structures, except they found the ring to be slightly bent, rather than planar, in the *T*_1_(*n*, π*) state.

Fišanová’s computational result is supported by the *T*_1_(*n*, π*) ← *S*_0_ cavity ringdown (CRD) absorption spectrum of 2CP. We reported this room-temperature spectrum in 2003,^9^ prior to our PE^5^ and REMPI jet-cooled investigations. The room-temperature CRD spectrum shows progressions and sequence bands involving ν_30_ (out-of-plane ring bending or puckering), the lowest-frequency mode of the molecule. A fitted one-dimensional ν_30_ potential for the *T*_1_(*n*, π*) state yielded a ring-bent equilibrium geometry with a barrier to planarity of about 40 cm^–1^.^9^

Figure 2 shows minimum-energy structures for the ^3^(*n*, π*) and ^3^(π, π*) states, calculated in the present investigation using the equation-of-motion excitation energies coupled cluster singles and doubles (EOM-EE-CCSD) technique. These structures are similar to the ones obtained previously^4,6,7^ at lower levels of theory. At near-planar geometries, the ^3^(π, π*) state is higher in energy than ^3^(*n*, π*). Throughout this paper, we refer to ^3^(π, π*) as *T*_2_ to reflect this ordering, though the ^3^(π, π*) state becomes lower than ^3^(*n*, π*) at “relaxed”^2^ ring-twisted geometries.

**Figure 2:**
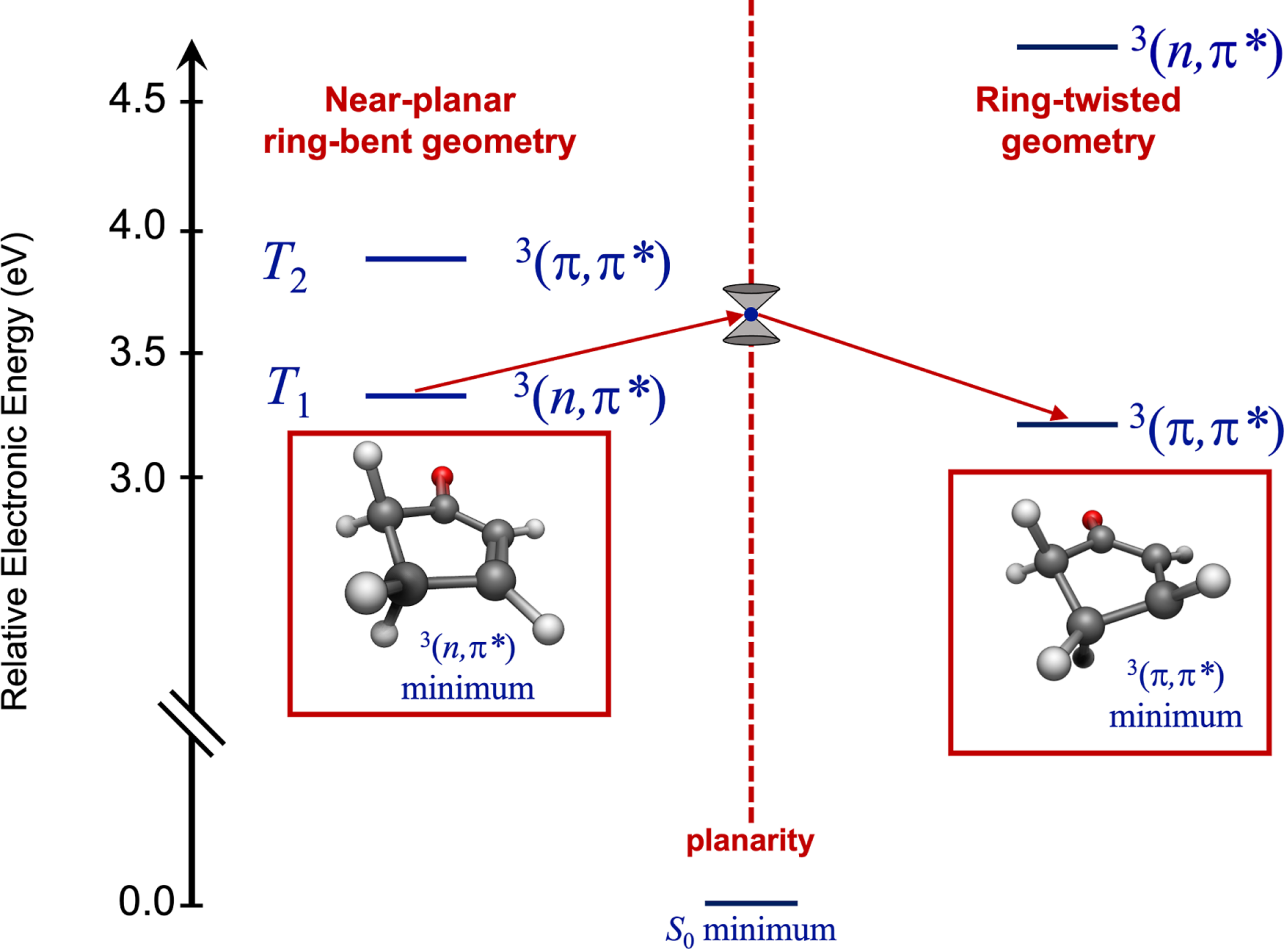
Minimum-energy structures for the lowest ^3^(*n*, π*) and ^3^(π, π*) states of 2CP, determined in this work via EOM-EE-CCSD/cc-pwCVTZ calculation. The double-cone icon in the center of the figure marks the position of the ^3^(*n*, π*)/^3^(π, π*) conical intersection calculated by García-Expósito et al.^4^ using CASSCF. Arrows show schematically the transition from a near-planar to ring-twisted geometry on the lowest triplet surface. The labels *T*_1_(*n*, π*) and *T*_2_(π, π*) reflect the ordering of states at near-planar geometries in the optically accessible *S*_1_(*n*, π*) ← *S*_0_ Franck–Condon (FC) region. Adapted from ref 4. Copyright 2001 American Chemical Society.

In addition to finding equilibrium geometries of the excited states, García-Expósito et al.^4^ used CASSCF to locate the minimum-energy crossing point between *S*_1_(*n*, π*) and *T*_1_(*n*, π*), as well as a minimum-energy ^3^(*n*, π*)/^3^(π, π*) conical intersection. The latter occurs at a planar geometry and is marked in Figure 2 at its CASSCF-calculated energy, relative to the ^3^(π, π*) minimum. The surface crossings are accessible when the *S*_1_(*n*, π*) state is prepared optically in the *S*_1_(*n*, π*) ← *S*_0_ FC region. The ^3^(π, π*) state, populated nonradiatively at a planar geometry, facilitates photochemistry on the triplet surface. The ^3^(π, π*) state can also relax via twisting about the C=C bond. Ensuing photophysics occurs at a ring-twisted geometry on the ^3^(π, π*) surface. In this region of the potential, a crossing between the ^3^(π, π*) state and a high-energy region on *S*_0_ causes relaxation to the ground state.^4^

The mapping of critical points on the 2CP Born–Oppenheimer surfaces set a foundation for computational work on the nonadiabatic dynamics of the relaxation. In 2021, Mukherjee and Varganov^8^ used the CASSCF method in a generalized ab initio multiple spawning (GAIMS) technique to model the relaxation dynamics of 2CP following vertical excitation to *S*_1_(*n*, π*). They found that the *S*_1_(*n*, π*) state decays promptly by ISC to *T*_1_(*n*, π*) and *T*_2_(π, π*) at a planar geometry, consistent with surface intersections calculated by García-Expósito et al.^4^ However, the GAIMS dynamics simulations do not identify a ring-twisted geometry for the ^3^(π, π*) state in the decay pathway. This structure is expected to mediate ISC to the ground state, according to the single-point CASSCF calculations of García-Expósito et al. The discrepancy in the two investigations could be attributed to a difference in the state-averaging protocols used for CASSCF.

Techniques such as GAIMS can predict the general features of a nonadiabatic decay pathway but are limited in precision because the chosen ab initio method must be relatively inexpensive. To obtain high-quality trajectories, it is necessary to know the shapes of the relevant excited-state potentials in detail, so that the crossing points can be calculated correctly. The CASSCF method does not quite meet this standard because it does not include dynamical correlation. To improve the prediction of nonadiabatic dynamics, it is desirable to identify an excited-state computational method with an accuracy/cost ratio greater than that of CASSCF.

Spectroscopic data, such as those we report here, can support this goal. From the newly recorded *T*_1_(*n*, π*) ← *S*_0_ REMPI spectrum of 2CP, we have extracted fundamental frequencies for eight vibrational modes in the *T*_1_(*n*, π*) state, including four modes that were not observable in our previous^5,9^ spectroscopic work. Measured vibrational frequencies are critical for testing the accuracy of computed excited-state potentials. Identifying computational methods with an optimal accuracy/cost ratio can ultimately broaden the reach of excited-state dynamics calculations for photochemically important molecules such as 2CP.

In this paper, we use the observed *T*_1_(*n*, π*) fundamental frequencies of 2CP to validate a computational approach, termed coupled-cluster/density functional theory (CC/DFT) hybrid, that was developed by Puzzarini et al.^10^ and Barone et al.,^11^ for predicting spectroscopic properties of medium-sized organic^11^ or biomolecules.^10,12^ This method utilizes the coupled cluster singles and doubles with perturbative triples (CCSD­(T)) ab initio method to determine harmonic frequencies, with anharmonic contributions obtained by DFT. In 2021, Sheng et al.^12^ successfully applied the CC/DFT method to the electronic ground state of serine, a conformationally flexible molecule with one more heavy atom than 2CP. In the present investigation of 2CP, we apply the CC/DFT method for the first time to an excited electronic state.

Though it was developed for electronic ground states, the CC/DFT approach is applicable to the *T*_1_(*n*, π*) excited state of 2CP because both the CC and DFT components of the calculations begin with a variational (SCF) construction of molecular orbitals. The variational approach converges to the lowest-energy state of a given spin multiplicity (neglecting spin–orbit coupling). The lowest-energy triplet state of 2CP at a near-planar geometry is the *T*(*n*, π*) state that we observe spectroscopically. We were able to carry out the CC/DFT calculations of this state using standard implementations of CCSD­(T) and DFT, along with an unrestricted Hartree–Fock (UHF) reference state.

We conclude the paper by using experimentally validated CC/DFT predictions to benchmark more economical approaches for determining excited-state frequencies. To this end, we employed EOM-EE-CCSD and time-dependent density functional theory (TDDFT) to calculate *T*_1_(*n*, π*) harmonic frequencies within the hybrid scheme, while continuing to calculate anharmonic corrections using unrestricted DFT. We compare the resulting *T*_1_(*n*, π*) fundamental predictions with CC/DFT reference values.

Such testing is especially important for medium-sized molecules such as 2CP. Most of the data sets for benchmarking frequency predictions have included only diatomics and small (noncyclic) polyatomic molecules.^13–15^ Moreover, very few of these investigations^16^ have examined excited-state computational methods. An important goal of this paper is to identify methods that are both generally applicable to excited states and less costly than CCSD­(T) for predicting vibrational frequencies. Such methods could potentially add insights into the current understanding of 2CP excited-state dynamics because they could treat not only the *T*_1_(*n*, π*) state but also *T*_2_(π, π*) and *S*_1_(*n*, π*). All three excited states are central to the photophysics and photochemistry of the 2CP molecule.

## Experimental and Computational Details

### Experiment

In this investigation, we used a one-color, (1 + 2) REMPI technique to record the *T*_1_(*n*, π*) ← *S*_0_ excitation spectrum of 2CP, with wavelengths ranging from 387 to 372 nm (3.2 to 3.3 eV). The (1 + 2) REMPI approach provides the requisite photon energy to reach the ionization threshold^17^ at 9.3 eV. The first photon is resonant with a vibronically resolved *T*_1_(*n*, π*) ← *S*_0_ transition. Two-photon absorption from the prepared *T*_1_(*n*, π*) level causes ionization. The photoionization is understood^18^ to proceed through an intermediate state that lies within a near-continuum of valence and Rydberg levels.

In our experiment, a liquid sample of 2CP is contained in a glass-wool plug inside a stainless-steel tube. The tube is installed within the vacuum chamber and heated to approximately 75 °C. The sample vapor is entrained in 2.0 bar of helium and expands supersonically into vacuum through a pulsed valve (Parker Series 9) placed directly after the heated tube. The valve body is heated with the same heating rope as the tube to prevent sample condensation prior to the expansion. The valve operates at 10 Hz and has a nozzle orifice of 0.5 mm. The free-jet expansion intersects with a 10 Hz pulsed laser approximately 10 cm downstream of the nozzle.

To detect ions created in the laser interaction region, we use a linear time-of-flight apparatus fabricated by Jordan TOF Products, Inc. The system has an ion-source component installed at the intersection of the laser beam and jet expansion. The ion source consists of extraction and acceleration grids as well as two sets of steering plates that direct the ions into a field-free time-of-flight tube of 1 m length. The mass-separated ions exit the tube and are detected by a “Z-Stack” microchannel plate (MCP) detector. The resulting time-of-flight mass spectrum, displayed on a digital oscilloscope, has a resolution of about 150. To capture the ion signal at a given excitation wavelength, we use an oscilloscope to average the parent-mass peak (82 amu) over 128 laser shots. We then send the digitized peak to a computer, where the peak area is calculated. The REMPI spectrum is a plot of the parent-ion peak area as a function of the laser excitation wavelength. The wavelength increment within a spectrum is typically 0.005 nm.

The excitation source is a Nd:YAG-pumped dye laser operating with LDS 751 dye at 10 Hz. The performance characteristics and wavelength calibration of the laser system have been described in detail elsewhere.^19^ Briefly, the dye laser produces 25 mJ output pulses at 760 nm with a bandwidth of about 0.1 cm^–1^. The output beam is sent through an angle-tuned β-(barium borate) doubling crystal, producing 380 nm pulses having an energy of 2–3 mJ. This beam is focused with a biconvex lens of focal length 50 cm, placed just outside of the vacuum chamber and about 35 cm away from the free-jet expansion. The focusing is needed to meet the fluence demands of the spin-forbidden singlet–triplet excitation, as well as the subsequent 2-photon ionization process.

The free-jet (i.e., skimmerless) expansion and ion extraction occur inside a source chamber consisting of a 6-way cross pumped by an Edwards Diffstak 100 diffusion pump. This pump has an internal water-cooled baffle. A separate baffle, cooled by a circulating ethylene glycol/water mixture at −10 °C, is located between the diffusion pump and vacuum chamber. This arrangement prevents the ion grids from being contaminated by backstreaming diffusion-pump fluid, but the external baffle reduces the effective pumping speed of the diffusion pump by about a factor of 2. As a consequence, the ambient pressure inside the source chamber is relatively high when the pulsed valve operates under normal conditions of 10 Hz and 2 bar of stagnation pressure. Under these conditions, the time-averaged pressure is about 2 × 10^–5^ Torr (2.7 × 10^–3^ Pa).

The MCP detector region is pumped separately from the source chamber. A turbomechanical pump (Leybold Turbovac 250 (i)) mounted near the MCP detector maintains a pressure of about 1 × 10^–6^ Torr (1.3 × 10^–4^ Pa) in this region while the pulsed valve is operating. Differential pumping occurs via a plate with a 1 cm hole that separates the source chamber from the time-of-flight tube.

### Computational Methods

We used the CFOUR 2.1 ^20^ computational chemistry package to carry out unrestricted CCSD­(T) calculations of the 2CP *T*_1_(*n*, π*) state. We used Gaussian 16 ^21^ for unrestricted DFT calculations and Q-chem 5.2 ^22^ for EOM-EE-CCSD and TDDFT calculations.

In preliminary work, we carried out geometry optimizations of the lowest ^3^(*n*, π*) and ^3^(π, π*) states of 2CP, using the EOM-EE-CCSD method with the cc-pwCVTZ^23^ basis set (see Figure 2). All electrons were correlated in these calculations; that is, we did not use the frozen-core (fc) approximation. The weighted cc-pwCVTZ basis set has been optimized^23^ to bias core–valence correlation effects over core–core correlation, resulting in smoother convergence to the complete basis-set (CBS) limit.

To aid vibronic assignments of the *T*_1_(*n*, π*) ← *S*_0_ REMPI spectrum, we conducted geometry optimizations and harmonic-frequency calculations of the *T*_1_(*n*, π*) state using unrestricted CCSD­(T)/pwCVTZ (all-electron), unrestricted DFT/def2-TZVP, and TDDFT/def2-TZVP techniques. The DFT-based calculations employed the Perdew, Burke, and Enzerhof hybrid functional without adjustable parameters (PBE0).^24^ Polarization functions in the def2/TZVP basis set are optimized for DFT calculations.^25^

We used *T*_1_(*n*, π*) fundamental frequencies extracted from the REMPI spectrum to validate the CC/DFT^10,11^ hybrid method. For the CC component of this procedure, we started with the CCSD­(T)/pwCVTZ harmonic frequencies. We extrapolated these values to the complete-basis set limit by conducting MP2/cc-pVTZ, MP2/cc-pVQZ, and MP2/aug-cc-pVTZ harmonic-frequency calculations, all within the fc approximation. Anharmonic force fields for the CC/DFT procedure were calculated using unrestricted DFT. We used the PBE0 functional, Becke’s three-parameter exchange functional with Lee–Yang–Parr correlation functional (B3LYP),^26^ the B3LYP functional corrected by the Coulomb-attenuating method (CAM-B3LYP),^27^ and the Grimme double hybrid functional with perturbative second-order correction (B2PLYP),^28^ all with the def2/TZVP basis set.

We investigated variants of CC/DFT to predict *T*_1_(*n*, π*) fundamentals more economically. These variants employ alternatives to CC harmonic-frequency calculations. In one approach, we selected EOM-EE-CCSD/cc-pVTZ (fc) for the harmonic part, and in others, we used TDDFT, with the B3LYP, CAM-B3LYP, or PBE0 functional.

We employed the WebMO graphical interface program^29^ to visualize and arrive at descriptions of the 2CP normal modes and to render isosurfaces of the molecule’s canonical molecular orbitals to confirm that the unrestricted calculations converged to the *T*_1_(*n*, π*) state.

## Results

### Vibronic Analysis of the Resonance-Enhanced Multiphoton Ionization Spectrum

Figures 3 and 4 show the *T*_1_(*n*, π*) ← *S*_0_ band system of 2CP, recorded using the (1 + 2) REMPI technique in a free-jet expansion. Wavenumber values are relative to the *T*_1_(*n*, π*) ← *S*_0_ 0_0_^0^ band at 25956.3 cm^–1^. Table 1 lists the positions and vibronic assignments of the REMPI bands. Also shown in the figures are two measurements of this band system we reported previously, the jet-cooled PE spectrum^5^ and the room-temperature CRD absorption spectrum.^9^ In our analysis of the CRD spectrum, we assigned the origin band (0_0_^0^) of the *T*_1_(*n*, π*) ← *S*_0_ transition, along with numerous vibronic bands involving the ring puckering mode, ν_30_. We also assigned fundamental (*N*_0_^1^) bands of three other modes, up to about +450 cm^–1^ with respect to the origin. These fundamentals (Table 1 and Figure 3) appear with a high signal-to-noise ratio in the REMPI spectrum.

**Figure 3:**
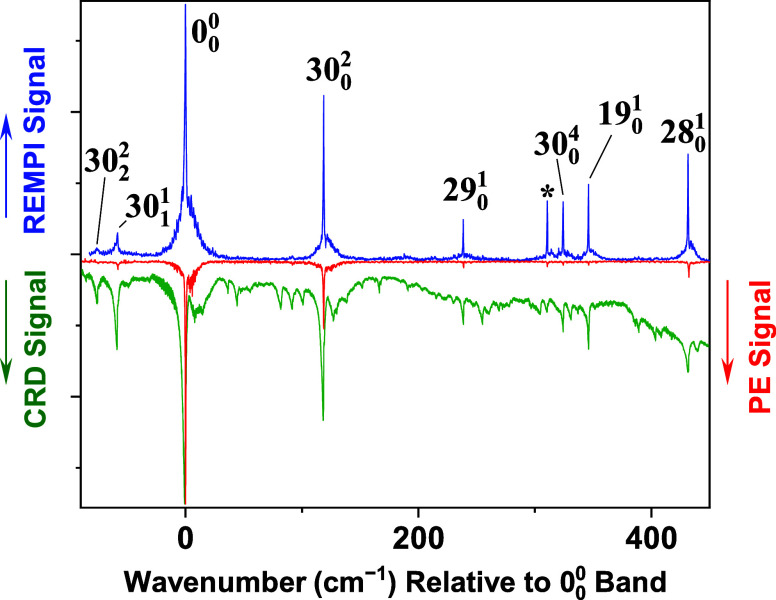
Upper trace: (1 + 2) REMPI spectrum of the *T*_1_(*n*, π*) ← *S*_0_ transition of 2CP, recorded under jet-cooled conditions. Wavenumber values are relative to the origin band at 25956.3 cm^–1^. The asterisk marks a band tentatively assigned^5^ to the lowest-energy *T*(π, π*) ← *S*_0_ system. Lower traces: room-temperature CRD and jet-cooled PE spectra of 2CP, reproduced from refs 5 and 9 , respectively. The CRD spectrum was recorded using 1.3 Torr of sample vapor in a 1 m cell. All three spectra have been scaled vertically so that the origin band has the same peak height.

**Figure 4:**
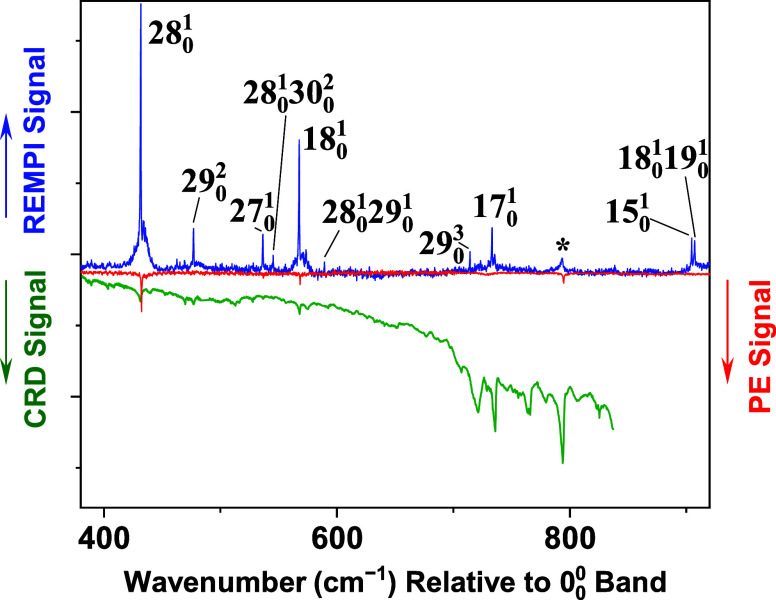
Continuation of the spectra of Figure 3 to about +900 cm^–1^ with respect to the *T*_1_(*n*, π*) ← *S*_0_ origin. Compared to Figure 3, the REMPI and PE spectra have been scaled up vertically by a factor of 2.5; the CRD spectrum has been scaled down vertically by a factor of 2.5. The feature marked with an asterisk is assigned to the *S*_1_(*n*, π*) ← *S*_0_ system. Details of this assignment are in the text.

**Table 1:** Vibronic Band Positions^a^ in the *T*_1_(*n*, π*) **←** *S*_0_ REMPI Spectrum of 2CP

observed pos. (cm^–1^)	inferred^b^ pos. (cm^–1^)	assignment	calculation of inferred pos. (cm^–1^)	intensity
–76.1^c^	–75.5	30_2_^2^ ^d^	118.6–194.1^e^	VW
–58.3		30_1_^1^ ^d^		M
0.0		0_0_^0^		VS
118.6		30_0_^2^ ^d^		S
238.5		29_0_^1^		M
324.3		30_0_^4^ ^d^		M
346.0		19_0_^1^		M
431.6		28_0_^1^		S
462.6	464.6	19_0_^1^30_0_^2^	346.0 + 118.6	VW
476.9	477.0	29_0_^2^	2 × 238.5	M
536.5		27_0_^1^		M
545.3	550.2	28_0_^1^30_0_^2^	431.6 + 118.6	W
567.6		18_0_^1^		M
589.2	584.5	28_0_^1^29_0_^1^	346.0 + 238.5	VW
714.2	715.5	29_0_^3^	3 × 238.5	W
733.3		17_0_^1^		M
904.6		15_0_^1^		M
907.2	913.6	18_0_^1^19_0_^1^	567.6 + 346.0	M

a Listed band positions are maxima relative to the 0_0_^0^ origin-band maximum, observed at 25956.3 cm^–1^.

b The locations of certain overtones, sequences, and combination bands are predicted assuming additivity of other REMPI band positions in this table.

c Uncertainty in a band maximum, due to noise at the tops of the peaks, is ±0.3 cm^–1^.

d This band was previously observed in the CRD spectrum^9^ and assigned as part of the fitting procedure for the anharmonic (double-well) ring-bending potential in the *T*_1_(*n*, π*) state.

e Ground-state level is from the far-infrared spectrum of 2CP vapor.^31^

We made the original CRD assignments by assuming the *T*_1_(*n*, π*) frequencies are similar to those established for the *S*_1_(*n*, π*) state from the *S*_1_(*n*, π*) ← *S*_0_ fluorescence excitation spectrum.^30^ We then secured the *T*_1_(*n*, π*) ← *S*_0_ assignments by locating hot bands (*v*″ > 0) in the CRD spectrum at positions predicted by ground-state combination differences. Ground-state frequencies of 2CP were obtained from the molecule’s gas-phase infrared spectrum.^31^

Both the jet-cooled REMPI and PE spectra in Figure 3 show previously assigned CRD cold bands as isolated sharp peaks, free from hot-band congestion. The vibronic bands in the REMPI spectrum have rotational contours that are broader than those in the PE spectrum. This could be due to a higher effective rotational temperature in the expansion. The pumping speed in the REMPI experiment is lower (see Experimental and Computational Details), and therefore, a higher fraction of each gas pulse is thermalized by collisions with the chamber walls before being pumped out. In the REMPI experiment, this creates an ambient background of room-temperature molecules that could be excited with each laser pulse in addition to the cold molecules in the supersonic expansion. Both populations are sampled by the laser, causing a broader rotational profile than that in the absence of room-temperature molecules.

Another contributor to the broader bandwidths in the REMPI spectrum could be saturation broadening. The laser pulse energy used for the REMPI experiment is about twice that of the PE experiment,^5^ and in the former, the laser is gently focused into the jet-expansion region. The high laser fluence is needed to meet the demand of 2-photon ionization detection.

At wavenumber values greater than +450 cm^–1^ in the REMPI spectrum (Figure 4) are vibronic bands that were not assignable in our previous work. These features are obscured by congestion in the room-temperature CRD spectrum and have insufficient emission quantum yield to be detected with certainty in the jet-cooled PE spectrum. The jet-cooled REMPI technique overcomes these impediments. To assign the newly observed REMPI bands and to confirm previous assignments in the lower-energy region of the *T*_1_(*n*, π*) ← *S*_0_ spectrum, we carried out *T*_1_(*n*, π*) harmonic-frequency calculations using three different methods: unrestricted CCSD­(T), unrestricted DFT, and TDDFT. We employed the PBE0 density functional for the DFT-based calculations. In previous investigations of related cyclic enone molecules,^19,32^ we found the PBE0 functional to be especially accurate for calculating excited-state vibrational frequencies.

Table 2 lists computed harmonic frequencies for the *T*_1_(*n*, π*) state, along with fundamentals obtained from *N*_0_^1^ vibronic band positions in the REMPI spectrum. The measured ground-state fundamentals^31^ are given for reference.

**Table 2:** Vibrational Frequencies (cm^–1^) of 2CP

		obs. fundamental		*T*_1_(*n*, π*) harmonic calculation		
mode no.^a^	description	*S*_0_ ^b^	*T*_1_(*n*, π*)^c^	U-CCSD(T)^d^	U-DFT^e^	TDDFT^e^
30	oop ring bend (pucker)	95	36.4^f^	74	21	70
29	ring twist	287	238.5	240	243	248
28	oop carbonyl wag	532	431.6	418	425	435
27	oop β-CH wag	750	536.5	517	504	550
19	ip carbonyl wag	464	346.0	341	346	354
18	carbonyl scissors	630	567.6	593	597	598
17	ip ring bend	753	733.3	746	766	755
15	CH_2_CH_2_CH sym. stretch	912	904.6	922	931	938

a The *T*_1_(*n*, π*) state has a slightly ring-bent equilibrium geometry (*C*_1_ point group). However, we retain the mode numbering and description of the planar (*C*_s_) ground state to simplify the labeling of *T*_1_(*n*, π*) ← *S*_0_ vibronic transitions.

b From ref 31.

c From the *N*_0_^1^ band assignment in the REMPI spectrum, except where noted.

d cc-pwCVTZ basis set.

e PBE0 functional with def2-TZVP basis set.

f The 30_0_^1^ band does not appear in the REMPI spectrum, but the 30_1_^1^ band does. We inferred the ν_30_^′^ fundamental by adding the ν_30_^″^ fundamental of 94.7 cm^–1^ (from the far-infrared spectrum^31^) to the position of the 30_1_^1^ band measured in the REMPI spectrum.

Agreement among the three excited-state computational methods is very good; for a given *T*_1_(*n*, π*) normal mode, each calculation returned nearly the same set of atomic displacement vectors, with frequency predictions typically varying by less than 20 cm^–1^ among the methods. For the most part, the *T*_1_(*n*, π*) harmonic calculations predict frequency values slightly greater than the observed fundamentals; this outcome can be reasonably attributed to the neglect of anharmonicity in the calculations. (Later, when we compare the performance among various computational models, we detail CC/DFT^10,11^ calculations that do incorporate anharmonicity.) For the lowest-energy (less than 500 cm^–1^) modes, the computed *T*_1_(*n*, π*) harmonic frequencies agree with observed fundamentals to within about 10 cm^–1^, except for the highly anharmonic (double-well) ν_30_ ring-puckering mode.

Bands involving ν_30_ were observed previously in the room-temperature CRD spectrum^9^ and assigned as part of our fitting procedure for the ring-puckering potential. Members of the 30_0_^*v*^ progression are irregularly spaced at low *v* ^33^ because this mode has a double-well potential in the upper state, with a low barrier that is located between the *v* = 0 and *v* = 1 levels. Even-numbered overtones of ν_30_^′^ are observed in the REMPI spectrum, as well as the 30_1_^1^ and 30_2_^2^ sequence bands. Transitions for which Δ*v*_30_ = odd are not apparent. These transitions have vanishingly small FC factors due to the near-planarity of the ring atoms in the *T*_1_(*n*, π*) state; in the planar limit (*C*_s_ point group), the ν_30_ mode would have *a*″ symmetry, as in the ground state, and the FC factor for Δ*v*_30_ = odd transitions would be zero. The 30_1_^1^ and 30_2_^2^ hot bands are relatively intense in the room-temperature CRD spectrum but have much diminished intensity under the jet-cooled conditions of the REMPI experiment.

For modes other than ν_30_ (i.e., higher-frequency modes), the jet-cooled REMPI spectrum contains *N*_0_^1^ transitions but no *T*_1_(*n*, π*) ← *S*_0_ hot bands. We do observe several overtones and combination bands involving higher-frequency modes (Table 1). We readily assigned these bands based on the corresponding fundamental frequencies.

Three of the *N*_0_^1^ transitions—29_0_^1^, 28_0_^1^, and 27_0_^1^—involve out-of-plane modes, and their vibrational overlap integrals are zero in the *C*_s_ limit. Nonetheless, these transitions appear with a relatively strong intensity in the *T*_1_(*n*, π*) ← *S*_0_ REMPI spectrum. They are made allowed by vibronic coupling involving nearby singlet excited states. The latter provide oscillator strength to the *T*_1_(*n*, π*) ← *S*_0_ transition via spin–orbit interaction with *T*_1_(*n*, π*). The strongest singlet contributor is likely *S*_2_(π, π*), according to El-Sayed’s rule.^34^ For an *N*_0_^1^ (*a*″) transition in the *T*_1_(*n*, π*) ← *S*_0_ system, the upper-state (*T*_1_, *v* = 1) level can spin–orbit couple efficiently to the (*S*_2_, *v* = 1) level, which has *A*′ × *a*″ = *A*″ vibronic symmetry. Transitions to this singlet level from the *v* = 0 ground-state level are FC-forbidden, but Herzberg–Teller coupling to an *A*″ singlet state (for example, *S*_1_(*n*, π*)) can facilitate the Δ*v* = 1 transition—ultimately providing the oscillator strength for such transitions in the *T*_1_(*n*, π*) ← *S*_0_ system.^35^

The REMPI spectrum contains a sharp peak at 26,267 cm^–1^ (asterisk in Figure 3) that cannot be assigned to a vibronic transition within the *T*_1_(*n*, π*) ← *S*_0_ system. The unassigned peak is also observed as a sharp feature in the jet-cooled PE spectrum^5^ but as a relatively weak and broad band in the room-temperature CRD spectrum.^9^ We have attributed^5^ this band to the nearby *T*(π, π*) ← *S*_0_ system.[Fn fn1]

A REMPI feature at 26747.7 cm^–1^ (asterisk in Figure 4) is assigned as a hot band of the *S*_1_(*n*, π*) ← *S*_0_ electronic transition. The latter has its 0_0_^0^ band at 27,210 cm^–1^.^30^ The *S*_1_(*n*, π*) ← *S*_0_ transition is spin-allowed and has a much higher oscillator strength than that of the *T*_1_(*n*, π*) ← *S*_0_ transition. Thus, it is possible to detect vibronic hot bands of the *S*_1_(*n*, π*) ← *S*_0_ system even under jet-cooled REMPI and PE conditions. The band at 26747.7 cm^–1^ is 462 cm^–1^ to the red of the *S*_1_(*n*, π*) ← *S*_0_ origin, consistent with a 19_1_^0^ assignment. The ground-state ν_19_ fundamental was determined to be 464 cm^–1^ by far-infrared spectroscopy.^31^

The REMPI spectrum contains many intense *S*_1_(*n*, π*) ← *S*_0_ features at wavenumbers slightly higher than those in Figure 4. These bands are located near the *S*_1_(*n*, π*) ← *S*_0_ origin, which is about 1250 cm^–1^ above the *T*_1_(*n*, π*) ← *S*_0_ origin. Many of these *S*_1_(*n*, π*) ← *S*_0_ bands are subject to saturation broadening, given the high pulse energies required to observe the *T*_1_(*n*, π*) ← *S*_0_ bands via the (1 + 2) REMPI scheme. The resulting congestion makes it difficult to assign *T*_1_(*n*, π*) ← *S*_0_ bands at wavenumbers higher than about 900 cm^–1^ relative to the triplet origin. Work is underway to implement a two-color resonant two-photon ionization ([1 + 1′] R2PI) scheme to detect *T*_1_(*n*, π*) ← *S*_0_ transitions in this higher-energy region. The excitation pulse energy required for this scheme is significantly lower than that of the one-color (1 + 2) method. This will lead to much narrower *S*_1_(*n*, π*) ← *S*_0_ features that should be distinguishable from *T*_1_(*n*, π*) ← *S*_0_ bands on the basis of rotational contours.

### Computational Results

We used the *T*_1_(*n*, π*) fundamentals assigned in the REMPI spectrum to validate the CC/DFT^10,11^ hybrid method for calculating these frequencies.

For the harmonic (CC) part of the calculation, we employed an additivity scheme^10,12^ that produces the best theoretical estimate (BTE) of harmonic frequencies (ω_*i*_) in the *T*_1_(*n*, π*) state. The components of the calculation are listed in the headings of Table 3. The starting point is a CCSD­(T)/cc-pwCVTZ calculation, using a UHF reference and correlating all elections, that is, omitting the fc approximation. This is the highest level of theory for harmonic frequencies that is compatible with the resources of our high-performance computing cluster.

**Table 3:** Contributions (cm^–1^) to a Composite Scheme for Calculating BTE of 2CP Harmonic Frequencies in the *T*_1_(*n*, π*) State

mode no.	ω(CCSD(T)/cc-pwCVTZ, all^a^)	Δω(CBS)^b^	Δω(aug)^c^	ω (BTE)
29	239.9	–2.2	–4.1	233.6
28	418.4	6.6	–0.7	424.2
27	516.7	4.9	6.3	527.8
19	340.9	3.8	–1.4	343.2
18	592.7	4.1	–0.2	596.7
17	746.4	7.3	–0.7	753.0
15	922.4	2.1	–2.2	922.3

a All electrons were correlated.

b From eq 3.

c ω­(MP2/aug-cc-pVTZ,fc) – ω (MP2/cc-pVTZ,fc).

The CCSD­(T)/cc-pwCVTZ calculation of the *T*_1_(*n*, π*) state produces nonimaginary frequencies for all 30 vibrational modes of the 2CP molecule. This calculation gives ⟨*S* ^2^⟩ = 2.19, indicating a modest amount of spin contamination. (⟨*S* ^2^⟩ = 2.00 for a pure triplet state.) The spin contamination does not appear to compromise the agreement with the observed frequencies, as will be seen below. All ω_*i*_ values from the CCSD­(T)/cc-pwCVTZ calculation are provided in the Supporting Information; Table 3 lists a subset for modes that are active in the REMPI spectrum, except ν_30_, ring-puckering. The double-well potential of this mode makes it a very poor candidate for analysis via computed harmonic frequencies.

The above calculation is subject to a basis-set truncation error. We approximated the CBS limit of ω_*i*_ by using an empirical extrapolation model^12,37^ describing the dependence on the ordinal number (*X*) of the correlation-consistent basis set

1 $$\omega_{i}\left(X\right)=a_{i}+b_{i}X^{-3}$$

where *a*_*i*_ = ω_*i*_(∞), the CBS limit of ω_*i*_. Following the approach of Sheng et al.,^12^ we determined *a*_*i*_ at the MP2 level, by employing unrestricted MP2/cc-pVQZ and MP2/cc-pVTZ harmonic-frequency calculations, both carried out within the fc approximation. Substituting *X* = 4 and *X* = 3 into eq 1 and solving for *a*_*i*_ gives

2 $$\omega_{i}\left(CBS,\;MP2\right)=\left[\frac{4^{3}\omega_{i}\left(Q\right)-3^{3}\omega_{i}\left(T\right)}{4^{3}-3^{3}}\right]$$

where ω_*i*_(*Q*) and ω_*i*_(*T*) are MP2 results with cc-pVQZ and cc-pVTZ basis sets, respectively. From this result, we obtained a correction, Δω_*i*_(CBS), to apply to the CCSD­(T)/cc-pwCVTZ frequencies in Table 3:

3 $$\Delta \omega_{i}\left(CBS\right)=\omega_{i}\left(CBS,\;MP2\right)-\omega_{i}\left(T,\;MP2\right)$$

This extrapolation procedure is compromised by the relatively small values of *X* that we used. To refine the result,^12^ we computed an additional correction to the CCSD­(T)/cc-pwCVTZ frequencies, incorporating effects of diffuse basis-set functions. This term is Δω_*i*_ (aug), the difference in unrestricted MP2 (fc) harmonic frequencies when calculated with the aug-cc-pVTZ basis set compared to cc-pVTZ. For small biomolecules, this term has been used empirically^10,12^ to improve upon the extrapolated ω_*i*_(CBS) values used in the CC/DFT procedure. Table 3 lists the Δω_*i*_(CBS) and Δω_*i*_(aug) correction terms applied to the CCSD­(T)/cc-pwCVTZ harmonic frequencies to yield ω_*i*_(BTE) values for the *T*_1_(*n*, π*) state of 2CP.

For the anharmonic part of the CC/DFT procedure, we used unrestricted DFT to calculate the cubic and semidiagonal quartic force constants of the *T*_1_(*n*, π*) state. We selected three density functionals—B3LYP, CAM-B3LYP, and PBE0—that have given the best performance in unrestricted CC/DFT anharmonic frequency calculations^38^ of small, semirigid free radicals in their electronic ground states. We also chose the widely used B2PLYP double-hybrid functional. The def2-TZVP basis set was used for all of the DFT anharmonic calculations.

To predict *T*_1_(*n*, π*) fundamental frequencies within the CC/DFT approach, we employed second-order vibrational perturbation theory (VPT2), using harmonic functions with ω_*i*_ (BTE) eigenvalues as a zero-order basis. The anharmonic analysis procedure, as implemented in the Gaussian 16 computational package, evaluates perturbation (e.g., Fermi and Darling-Dennison) matrix elements via anharmonic force constants obtained by DFT. The Gaussian 16 code uses input frequencies (ω_*i*_(BTE) in our case) to identify near-resonant harmonic states. The VPT2 energies are deperturbed^39^ by removing large off-diagonal contributions associated with the resonances. The involved harmonic functions are redeployed in a subsequent variational treatment that diagonalizes a small anharmonic matrix explicitly.

We included all modes of 2CP except ν_30_ in this analysis. Inclusion of the highly anharmonic ring-puckering mode leads to unrealistically large off-diagonal terms in the perturbation matrix formed from the harmonic basis functions.

Table 4 lists vibrational frequencies obtained from the CC/DFT hybrid calculations of the 2CP *T*_1_(*n*, π*) state, along with experimental fundamentals extracted from the *T*_1_(*n*, π*) ← *S*_0_ REMPI spectrum. In the table, we also report the mean absolute error (MAE) for the listed vibrational modes. For a given mode, we define absolute error as the unsigned difference between the experimentally observed fundamental frequency and the value calculated by using the CC/DFT hybrid procedure.

**Table 4:** Accuracy of the CC/DFT^a^ Hybrid Procedure for Computing Fundamental Frequencies (cm^–1^) of 2CP in Its *T*_1_(*n*, π*) State

mode no.	B3LYP	CAM-B3LYP	PBE0	B2PLYP	exp.^b^
29	236.7	236.5	240.1	239.8	238.5
28	419.1	418.4	417.9	418.9	431.6
27	528.9	539.4	541.8	534.2	536.5
19	344.3	342.2	341.6	342.1	346.0
18	589.3	586.4	586.8	588.6	567.6
17	713.1	734.9	734.4	711.9	733.3
15	905.6	906.2	905.5	904.6	904.6
MAE^c^	9.5	6.3	6.6	8.9	—
MAPE^d^	1.7%	1.3%	1.4%	1.7%	—

a The listed density functionals were used with the def2-TZVP basis set to determine anharmonic force constants; VPT2 analysis incorporated the ω_*i*_(BTE) harmonic frequency values from Table 3.

b From the *T*_1_(*n*, π*) ← *S*_0_ REMPI spectrum.

c MAE, with respect to experimentally measured fundamentals.

d Mean absolute percent error, with respect to experimentally measured fundamentals.

The results in Table 4 show that the CC/DFT hybrid method predicts the *T*_1_(*n*, π*) fundamental frequencies of 2CP very accurately. The MAE value, averaged over the four chosen density functionals, is 7.8 cm^–1^. This result validates CC/DFT for an excited electronic state at the same level of accuracy as has been established for ground-state molecules. For example, Sheng et al.^12^ and Puzzarini et al.^10^ used the CC/DFT method to predict fundamental frequencies of serine and uracil, respectively, in their electronic ground states. They obtained MAEs of 8.5 cm^–1^ and 11 cm^–1^, respectively, which are comparable to the present MAE for the *T*_1_(*n*, π*) state of 2CP. The uracil and serine MAEs each incorporated about twice the number of experimental frequencies as available for 2CP; thus, a more complete assessment awaits measurement of additional 2CP *T*_1_(*n*, π*) frequencies. This should be feasible through planned two-color R2PI experiments.

The CAM-B3LYP and PBE0 functionals are particularly well suited for the present CC/DFT calculations. Percent errors for fundamentals are less than 1.5% when these functionals are used to calculate anharmonicity. The only significant performance problem for the CAM-B3LYP or PBE0 calculation occurs for ν_28_ (out-of-plane carbonyl wag). The CC/DFT frequency predictions for this mode are less than observed, not greater than, as would be expected^40^ if the error originated in the harmonic calculation. The underestimate could be due to the omission of ν_30_, the lowest-frequency mode, in the VPT2 analysis. Anharmonic coupling between ν_30_ and ν_28_ could cause a significant upward shift in the latter frequency, bringing it into closer agreement with the measured value.

Having validated the CC/DFT method for the 2CP *T*_1_(*n*, π*) state, we used its predictions to benchmark more economical approaches for calculating the fundamental frequencies. For the harmonic part, we considered EOM-EE-CCSD and TDDFT, two widely used methods designed specifically for excited electronic states. To calculate an anharmonic force field, we employed unrestricted DFT as before. We chose the CAM-B3LYP functional for this component of the calculation because it produces the lowest MAE in the CC/DFT calculation (Table 4). We incorporated the CAM-B3LYP anharmonic constants into a VPT2 calculation that uses EOM-EE-CCSD or TDDFT harmonic frequencies. We refer to these hybrid approaches as EOM/DFT or TDDFT/DFT.

Table 5 presents the *T*_1_(*n*, π*) frequencies of 2CP obtained from the EOM/DFT and TDDFT/DFT calculations. The TDDFT/DFT frequencies are generally higher than CC/DFT reference values. This is expected when a DFT-based method rather than an ab initio calculation is used to determine harmonic frequencies. For one mode, ν_27_, the CAM-B3LYP/DFT and EOM/DFT frequencies both show extreme departures (+83 cm^–1^) from the CC/DFT reference values. In these cases, the order of the ν_27_ and ν_18_ harmonic frequencies is switched relative to the CC/DFT calculation. These switches led to large mean absolute deviations (MADs) from the reference values, greater than 20 cm^–1^, for two entries in Table 5. However, the MAD values among the TDDFT methods even out to about 17 cm^–1^ when they are calculated over 23 vibrational frequencies of 2CP under 1500 cm^–1^, corresponding to the non-C–H stretch vibrations. These frequencies are provided in Supporting Information, and MADs are listed in the final row of Table 5. It is notable that the more expensive EOM-EE-CCSD ab initio method performs worse within this hybrid scheme than any of the TDDFT methods. We discuss the implications of this finding in the next section.

**Table 5:** Relative Performance of Various Hybrid^a^ Procedures for Computing Fundamental Frequencies (cm^–1^) of 2CP in Its *T*_1_(*n*, π*) State

			TDDFT^d^/DFT			
mode no.	CC^b^/DFT	EOM^c^/DFT	B3LYP	CAM-B3LYP	PBE0	exp.
29	236.5	226.2	263.7	208.6	251.2	238.5
28	418.4	413.7	437.6	426.7	429.3	431.6
27	539.4	622.4	537.5	621.6	562.1	536.5
19	342.2	330.1	357.2	346.2	353.4	346.0
18	586.4	575.9	590.2	580.9	587.0	567.6
17	734.9	706.2	735.7	725.1	737.3	733.3
15	906.2	923.5	902.4	921.7	922.8	904.6
MAE^e^	6.3	26.6	10.2	22.6	12.8	—
MAD^f^	—	23.8	10.0	21.9	11.3	—
MAD_all_ ^g^	—	19.8	16.7	17.7	17.2	—

a All calculations employed unrestricted DFT (CAM-B3LYP/def2-TZVP) to determine the anharmonic force field, with harmonic frequencies calculated by methods in the column headings. Results for all 30 modes of 2CP are provided in the Supporting Information.

b ω_*i*_(BTE) harmonic frequencies from Table 3 were used for the hybrid calculation.

c EOM-EE-CCSD/cc-pVTZ (fc) harmonic frequencies were used for the hybrid calculation.

d TDDFT/def2-TZVP harmonic frequencies were used for the hybrid calculation.

e MAE, with respect to experimentally measured fundamentals.

f MAD, with respect to the CC/DFT calculation, including only modes listed in this table.

g MAD, including all modes with frequency less than 1500 cm^–1^, except ν_30_ (ring pucker).

## Discussion

The high-level unrestricted CCSD­(T) ab initio technique forms the backbone of the computational study of 2CP presented here, and the CCSD­(T) results provide insights about the nature of the *T*_1_(*n*, π*) ← *S*_0_ chromophore. These insights can help rationalize the observed changes in vibrational frequencies upon excitation. Our improved understanding of the chromophore also helps to interpret the success or failure of various hybrid techniques for computing the *T*_1_(*n*, π*) fundamentals.

The *T*_1_(*n*, π*) equilibrium geometry obtained from the CCSD­(T) calculation is shown in Figure 5. Atomic Cartesian coordinates are provided in the Supporting Information. We carried out the geometry optimization with all electrons correlated, using an appropriately large (cc-pwCVTZ) basis set. Thus, for 2CP, the *T*_1_(*n*, π*) geometry presented here is one of the most accurate available within a modern high-performance computing environment.

**Figure 5:**
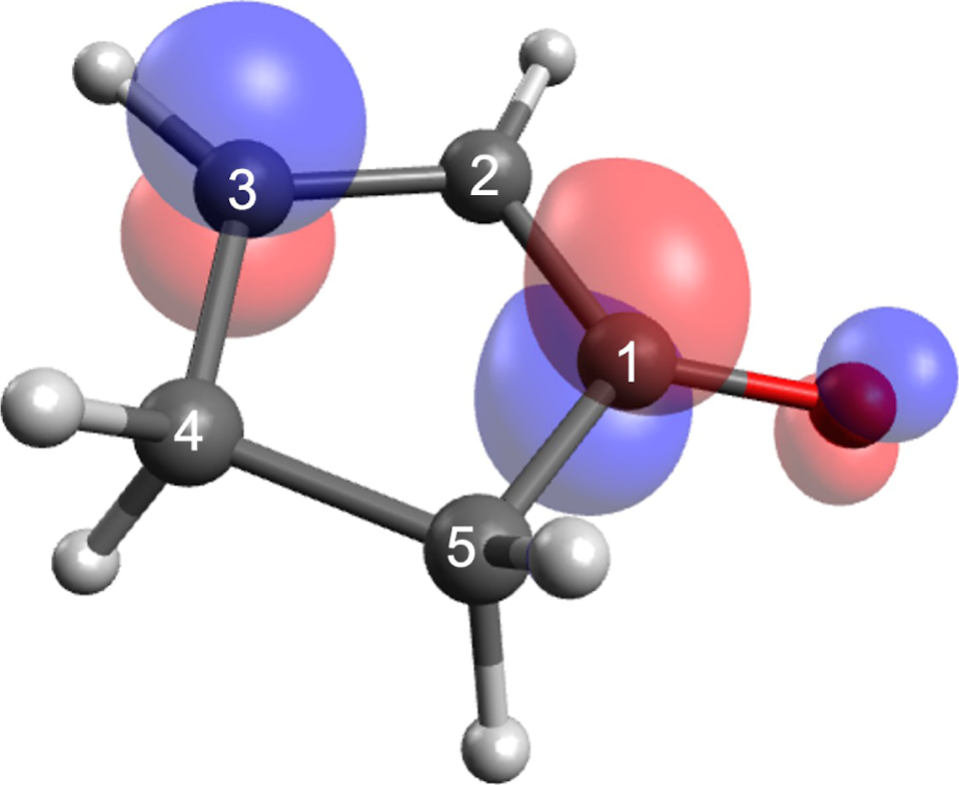
Structure of the *T*_1_(*n*, π*) state of 2CP, optimized via unrestricted CCSD­(T)/cc-pwCVTZ calculation. The isosurface depicts the singly occupied canonical π* orbital in the *T*_1_(*n*, π*) state. The value of |ψ| on the surface shown is 0.10 Å^–3/2^.

The ring structure in the *T*_1_(*n*, π*) state is slightly puckered, with a C_1_–C_2_–C_3_–C_4_ dihedral angle of −3.2°, according to the CCSD­(T) calculation. The nonplanarity stems from the promotion of a nonbonding electron into the π* LUMO. An isosurface of the latter orbital is shown in Figure 5. The electron density is localized mainly on C_3_ and C_1_, with a node in between. The nodal structure of the π* orbital reduces the C_2_–C_3_ bond order from its nominal value of 2 in the ground state. This allows the ring to pucker slightly, lowering the torsional strain associated with CH_2_ groups at positions 4 and 5. These groups are eclipsed in the electronic ground state but staggered by about 10° in the *T*_1_(*n*, π*) state.

The lowering of C_2_–C_3_ bond order in the *T*_1_(*n*, π*) state has a significant effect on the out-of-plane vibrational modes, that is, those classified as *a*″ in the ground state. Table 2 shows that these modes (ν_27_ through ν_30_) become less stiff upon *T*_1_(*n*, π*) ← *S*_0_ excitation. The drop in frequency is particularly dramatic for ν_27_ (out-of-plane β-CH wag) and ν_28_ (out-of-plane carbonyl wag). Both are delocalized into the ring and involve pyrimidalization at C_3_. This motion is eased in the excited state *T*_1_(*n*, π*) because it minimizes repulsion between the π* electron and σ bonds involving C_3_.

The in-plane (*a*′) ring modes ν_17_ (bending) and ν_15_ (stretching) undergo relatively small frequency reductions upon *T*_1_(*n*, π*) ← *S*_0_ excitation. The atomic displacements for the in-plane ring modes do not appreciably stabilize the π* antibonding electron.

The CC/DFT predictions of fundamental frequency in the *T*_1_(*n*, π*) state of 2CP (Table 4) are remarkably accurate, despite the complex interplay between stabilizing features of the molecule (e.g., conjugation and pentagonal ring geometry) and destabilizing features (torsional strain and occupancy of an antibonding orbital). Harmonic-frequency predictions at the CCSD­(T) level are expected to be systematically too high by 5–10 cm^–1^ in the CBS limit^40^ due to omission of higher-order connected excitations (e.g., *Q* and 5). Given that most of the errors (measured minus computed fundamental frequency) in Table 4 are in line with harmonic expectations, it appears that the CC/DFT method handles anharmonicity very accurately—particularly when the CAM-B3LYP and PBE0 functionals are used to calculate the anharmonic force constants. The other two functionals we tested, B3LYP and B2PLYP, produce slightly larger MAEs. This can be traced to overestimates of the anharmonic coupling strength for certain near-resonant levels.

Even with the B3LYP and B2PLYP anharmonic results included, the MAEs in Table 4 are all under 10 cm^–1^, with MAPEs under 2%. These are impressive statistics for fundamental-frequency calculations, particularly those involving an excited electronic state. Moreover, the direction and magnitude of the errors for the 2CP *T*_1_(*n*, π*) state are wholly consistent with those of prior CC/DFT investigations^10,38^ of similarly sized molecules in their ground states.

By contrast to CC/DFT results, the EOM/DFT and various TDDFT/DFT methods show a wide range of MAE in predicting *T*_1_(*n*, π*) fundamental frequencies, as seen in Table 5. Among the TDDFT methods, the CAM-B3LYP functional is the most problematic. It is possible that the long-range correction built into the CAM-B3LYP functional has an overcompensating effect in describing the potential near its equilibrium geometry. In the *T*_1_(*n*, π*) state of 2CP, this description must be very accurate to obtain the correct harmonic frequencies, given the delicately balanced influences of torsional strain, ring strain, and conjugation. The CAM-B3LYP functional performs much better (Table 4) when it is used in an unrestricted DFT calculation of anharmonicity. The long-range correction appears to be more beneficial in this case, given that the anharmonic part of the potential is more significant at geometries farther from equilibrium.

Finally, we note an unexpected outcome of the EOM/DFT calculation. Despite its higher cost, the EOM-EE-CCSD calculation of harmonic frequencies leads to the worst predictions of the *T*_1_(*n*, π*) fundamentals among the methods specified in Table 5. This result underscores the importance of including triple excitations in an ab initio frequency calculation. Very recently, Zhao and Matthews reported^41^ a promising development in this regard. They have obtained analytic gradients for EOMEE-CCSD*, an excited-state ab initio method that includes an approximate treatment of triple excitations at a cost slightly higher than that of EOM-EE-CCSD. Those authors are currently implementing EOMEE-CCSD* analytic gradients into the CFOUR package.^42^

## Conclusions

The 2CP molecule has attracted much interest within the computational photochemistry community because of the interplay of low-lying excited states populated after photoexcitation. A consistent theme of prior computational work^4,7,8^ is to describe how the lowest triplet excited states of 2CP mediate the photophysical decay pathways and the ensuing photochemistry of the molecule. The quality of these predictions hinges on an accurate calculation of the 2CP triplet-state potential surfaces. Experimental measurements provide critical feedback for refining such calculations.

In the present investigation, we have used REMPI spectroscopy to measure fundamental vibrational frequencies in the *T*_1_(*n*, π*) state of 2CP. The *T*_1_(*n*, π*) ← *S*_0_ REMPI spectrum extends the experimentally available^5,9^ *T*_1_(*n*, π*) frequency information from four to eight modes, now including fundamentals in the 500–900 cm^–1^ range. Half of the observed modes involve out-of-plane displacement of the ring atoms, and we find that these modes undergo frequency drops upon excitation that are more significant than those of the in-plane modes. This distinction has helped us understand how population of the π* antibonding orbital influences the structure and dynamics of the *T*_1_(*n*, π*) state of 2CP.

We used the experimental *T*_1_(*n*, π*) fundamental frequencies to evaluate the predictions from a variety of computational methods. At the core of our computational study is a high-level ab initio calculation of the *T*_1_(*n*, π*) harmonic frequencies. We employed the CCSD­(T) method for this calculation, with all electrons correlated, and extrapolated the result to the CBS limit. We then used the CC/DFT hybrid approach^10–12,38^ to obtain anharmonic fundamental frequencies for direct comparison to the *T*_1_(*n*, π*) fundamentals we measured experimentally. This work represents the first time, to the best of our knowledge, that CC/DFT has been applied to an excited electronic state. Computed frequencies for the *T*_1_(*n*, π*) state of 2CP are remarkably accurate, with errors of less than 10 cm^–1^ on average. These results for an excited state validate the CC/DFT method at the same level of accuracy as available for ground-state molecules of comparable size.

Given this success, we used the CC/DFT frequency predictions as a reference to evaluate alternative approaches for calculating *T*_1_(*n*, π*) frequencies. For photochemically relevant molecules such as 2CP, it is important to identify methods that are economical and generally applicable to excited states, not just the lowest state of a given spin multiplicity. To this end, we used the EOM-EE-CCSD and TDDFT excited-state techniques to calculate *T*_1_(*n*, π*) harmonic frequencies with anharmonicity incorporated as in the CC/DFT method.

The TDDFT harmonic calculations produced *T*_1_(*n*, π*) fundamentals deviating from reference values by 17 cm^–1^, on average. (This average considers all non-C–H stretch modes of the molecule.) The choice of functional is important—the popular B3LYP and PBE0 functionals performed best, particularly for the set of (low-frequency) modes represented in the REMPI spectrum. The EOM-EE-CCSD harmonic calculation was more prone to error, leading to deviations of 20 cm^–1^ on average from reference fundamental frequencies. This outcome highlights the significant influence of triple excitations in the CCSD­(T) reference calculation of the *T*_1_(*n*, π*) state.

In this context, it remains a challenge to identify accurate and economical methods for treating the excited states of 2CP in general. These states include *S*_1_(*n*, π*) and *T*_2_(π, π*), which are relevant to the molecule’s photochemistry. The TDDFT methods have been widely implemented, but the optimal choice of functional is not clear-cut, nor is the range of error to expect. We aim to narrow these uncertainties via planned two-color R2PI studies of the *T*_1_(*n*, π*) ← *S*_0_ system in higher-frequency regions of the spectrum.

Overall, this investigation has shown that it is possible to obtain very accurate *T*_1_(*n*, π*) fundamentals for 2CP using a hybrid computational approach with unrestricted CCSD­(T) at its core. This is a critical advance for benchmarking purposes, and it opens the door to accurate calculation of other properties, such as zero-point vibrational energy or crossing points with *T*(π, π*), that influence the photophysics of 2CP relaxation.

## Supplementary Material



## Footnotes

1 If the unassigned feature in Figure 3 is part of the *T*(π, π*) ← *S*_0_ system, the band broadening in the CRD spectrum at 1.3 Torr could be due to collision-induced *T* ⇝ *S*_0_ intersystem crossing (CIISC). This process is activated when the ground-state and triplet geometries are significantly different, as evidenced by phosphorescence yield studies of propynal.^36^ In 2CP, the ring-twisted equilibrium geometry of the *T*(π, π*) state could promote the CIISC and thereby lead to broadening of the unassigned feature in the CRD spectrum.

## Notes

This Excel document contains atomic coordinates of the 2CP *T*_1_(*n*, π*) geometry, optimized via CCSD­(T)/cc-pwCVTZ calculation. Also included are components of the ω­(BTE) calculation for the *T*_1_(*n*, π*) state as well as frequency results from the CC/DFT calculation and other hybrid methods (XLSX)

## §

Department of Chemistry, Brown University, United States

## ∥

Department of Chemistry, University of Southern California, United States
